# Bio-Based Viscoelastic Polyurethane Foams: Functional Behavior Across Application Temperatures

**DOI:** 10.3390/polym18020174

**Published:** 2026-01-08

**Authors:** Elżbieta Malewska, Konstantinos N. Raftopoulos, Piotr Rytlewski, Sławomir Michałowski, Natalia Koman, Maria Kurańska, Aleksander Prociak

**Affiliations:** 1 Faculty of Chemical Engineering and Technology, Cracow University of Technology, Warszawska 24, 31-155 Cracow, Poland; konstantinos.raftopoulos@pk.edu.pl (K.N.R.); slawomir.michalowski@pk.edu.pl (S.M.); natalia.koman@student.pk.edu.pl (N.K.); maria.kuranska@pk.edu.pl (M.K.); aleksander.prociak@pk.edu.pl (A.P.); 2 Faculty of Materials Engineering, Kazimierz Wielki University, Chodkiewicza 30, 85-064 Bydgoszcz, Poland; prytlewski@ukw.edu.pl

**Keywords:** polyurethane, viscoelastic foams, application temperature

## Abstract

Viscoelastic polyurethane foams were prepared using four different bio-based polyols derived from coconut oil (CO), palm oil (PO), duck fat (DF), and pork fat (PF), employing up to 20 wt.% of the polyol component in a conventional formulation. The introduction of bio-polyols into the polyurethane formulation gave rise to an early minor decomposition of modified foams at low temperatures; however, the overall thermal stability improved slightly by the elimination of some intermediate decomposition stages. The glass transition temperature of foams was only moderately influenced and remained in the typical temperature range (around 10 °C). The effect of biopolyol type and content (5–20 wt.%) on the mechanical properties of the foams was investigated over the temperature range −20 to 40 °C. At 20 and 40 °C, all foams exhibited comfortable viscoelastic properties suitable for furniture applications. Hysteresis and the damping behavior of foams were also influenced by biopolyol type and concentration, with CO and DF providing enhanced energy absorption. Overall, these bio-based foams demonstrate potential for eco-friendly, high-performance applications, although their use at temperatures below 10 °C may be limited by increased stiffness.

## 1. Introduction

Temperature is among the most critical parameters influencing the properties of materials, including polyurethane foams (PUFs). It affects material characteristics, not only during their use but also throughout the manufacturing process [1].

PUFs have been attracting increasing attention in recent years, and consequently, their range of applications continues to expand. This expansion involves exposure to a broader spectrum of operating temperatures. In light of this, it is essential to investigate the effect of application temperature on the behavior of PUFs. Such studies enable the precise selection of foam types for specific applications under defined temperature conditions, ensuring the preservation of their optimal properties.

Viscoelastic PUFs are a special type of flexible foam. Through careful selection of raw materials during foam synthesis, polyurethane develops a defined segmental structure that determines its mechanical behavior [2]. Viscoelastic foams show a significantly longer post-load recovery time (~1–20 s) than standard flexible PUFs. Such foams are commonly known as “shape memory foams,” even though their slow recovery behavior originates from viscoelasticity rather than a real shape memory mechanism [3]. This allows the foams to adapt to the shape of a body or object, providing comfort and support to users, and protecting objects from external forces, vibrations, or shocks. As a result, viscoelastic foams are used in outer layers of mattresses and pillows, including medical products, earplugs, prosthetic comfort inserts, shoe insoles, upholstered furniture, and shock-absorbing packaging for valuable or sensitive items [4,5,6]. In all these applications, the foams are exposed to different ambient temperatures as well as human body temperature. For example, packaging materials or winter shoe insoles may face subzero temperatures, while foams in contact with the human body are locally exposed to about 37 °C [1].

In the literature, studies most commonly address the effect of ambient temperature (Öchsner, 2008 [7]), mold temperature [8,9,10], and raw material temperature [11] on the polyurethane foaming process and, consequently, on the properties of foam materials such as cell structure, apparent density, and mechanical strength. All these characteristic temperatures have a significant impact on the chemical reactions involved in the formation of PUFs. During the foaming stage, polyurethane systems generally undergo gelling, foaming, and curing processes [12]. The higher the temperature during mixing and pouring of the polyurethane system, the greater the activity of the raw materials, namely isocyanate and polyol. In contrast, at lower temperatures, the reaction between the –NCO groups of isocyanates and the –OH groups of polyols is less efficient, which may result in poor foam quality [13].

Many authors have investigated the influence of ambient temperature on the performance of finished polyurethane materials. The majority of these studies, however, focus on rigid polyurethane foams, for which various mathematical models have been developed to predict their mechanical properties under specific temperature conditions [14]. The reviewed research collectively indicates that rigid polyurethane foams are highly sensitive to temperature, generally exhibiting a pronounced decrease in mechanical strength as temperature increases. For example, Horak et al. [15] and Song et al. [16] examined rigid foams of varying apparent densities under different thermal conditions and found that elevated temperatures resulted in reduced compressive and tensile strength, accompanied by a decrease in compressive modulus. In contrast, relatively few studies have addressed the functional and mechanical properties of flexible and viscoelastic polyurethane foams under different application environments.

The aim of this study was to evaluate the effect of ambient temperature in the range of −20 to +40 °C on selected physicomechanical properties of finished viscoelastic PUFs. In addition, different biopolyols were incorporated into the formulations to assess their influence on these performance characteristics. Biopolyols are renewable alternatives to conventional petrochemical polyols, derived from sources such as waste vegetable or animal fats. They are gaining increasing attention due to ongoing pro-ecological efforts aimed at reducing the consumption of fossil resources, lowering the carbon footprint of products, and decreasing greenhouse gas emissions. Nevertheless, biopolyols must also ensure that the resulting materials retain satisfactory performance properties.

Polyurethane foams obtained using the same bio-based polyols and prepared in an analogous manner were investigated in our previous publication, which focused primarily on the synthesis and chemical structure of the bio-polyols, as well as the properties of the resulting foams under room-temperature conditions [17]. The present manuscript constitutes a continuation of that research but addresses a distinct and complementary aspect by focusing on the temperature-dependent functional behavior of viscoelastic polyurethane foams across a broad range of application-relevant temperatures. This approach provides new insight into the performance of bio-based viscoelastic foams under practical service conditions, an aspect that remains rarely addressed in the available literature, and therefore represents a clear and original contribution beyond our earlier work.

## 2. Materials and Methods

The synthesis of the materials investigated in this study has been described elsewhere [17]. Briefly, viscoelastic PUFs were synthesized using the one-shot method. The base polyurethane system was prepared from the following raw materials: petrochemical polyols, biopolyols, a catalyst, a surfactant, water (as a chemical blowing agent), diethylene glycol (DEG) (as a chain extender), and an isocyanate. The formulation included a mixture of three different industrial petrochemical polyols (P1, P2, P3), characterized by hydroxyl numbers of 410, 33, and 29 mgKOH/g, respectively. The use of polyols with such distinct hydroxyl values within a single composition enabled the development of materials exhibiting viscoelastic properties, as the combination of highly functional and low-functional polyols allowed for controlled crosslink density and segmental mobility. This specific polyol blend was selected based on findings from our previous studies, which indicated that such a formulation provides an optimal balance between elastic recovery and time-dependent deformation [17]. The petrochemical polyols were supplied by PCC Rokita S.A. (Brzeg Dolny, Poland). Dibutyltin diacetate, provided by Evonik, was used as the catalyst, while Niax L617 (Momentive Performance Materials Inc., Niskayuna, NY, USA) served as the surfactant. Foaming was carried out using water as the chemical blowing agent, which reacts with isocyanate (NCO) groups to generate CO_2_. DEG was supplied by Chempur (Piekary Śląskie, Poland). The isocyanate component was Ongronat 3800 (4,4′-methylene diphenyl diisocyanate modified with carbodiimide), provided by BorsodChem Zrt. (Kazincbarcika, Hungary), with a free NCO group content of 28%.

The base system as described above was modified by four different bio-based polyols, incorporated into the polyurethane system at levels up to 20 wt.%. The selected range of bio-polyol content (5–20 wt.%) was based on preliminary formulation studies and literature data, indicating that this range enables partial replacement of petrochemical polyols without compromising foam processability or structural integrity. The bio-polyols were obtained from natural solid fats, namely coconut oil (CO), palm oil (PO), duck fat (DF), and pork fat (PF), which were purchased from a local retail store, via a transesterification process employing triethanolamine (TEA) as the transesterifying agent. The selection of CO-, PO-, DF-, and PF-based polyols was motivated by their availability, renewable origin, and significantly different fatty acid compositions, resulting in bio-polyols with diverse molecular structures. This diversity enables a systematic evaluation of the influence of biopolyol structure on the viscoelastic and thermal behavior of polyurethane foams. Moreover, these raw materials represent both plant- and animal-based feedstocks, allowing for a broader assessment of bio-based alternatives. Briefly, the oil/fat and TEA were reacted in a molar ratio of 1:3 at 175 °C for 2 h in the presence of zinc acetate (0.3%) as a catalyst. The reaction was carried out under stirring until the desired hydroxyl value was achieved, after which the product was cooled to room temperature and used without further purification. The detailed procedure and full characterization of the biopolyols have been described in previous publications [17,18,19]. Table 1 summarizes the key properties of the synthesized biopolyols, particularly hydroxyl number and water content, which significantly affect the formulation of the polyurethane system. The designation of each biopolyol was created by adding the prefix “bio” to the symbol of the oil or fat used for its synthesis.

To prepare the viscoelastic foams, the raw materials were weighed sequentially into a 0.5 L cup according to the specified formulation (Table 2). The polyol premix contained all formulation components without the isocyanate. The premix was stirred mechanically for 30 s. Subsequently, a defined amount of isocyanate, corresponding to an isocyanate index of 0.6, was added, and the mixture was stirred again for 5 s. The amount of isocyanate was adjusted according to the hydroxyl values of the applied polyols to maintain a consistent NCO/OH ratio across all formulations. This ensures comparable crosslinking density and validity of performance comparisons. The resulting blend was poured into a plastic mold and cured for 24 h. The amount of unreacted isocyanate was not examined, because a significant excess of polyol components was used in the formulation, as evidenced by the isocyanate index of 0.6. Therefore, the probability of the presence of unreacted –NCO groups is minimal. After 24 h, the foams were cut for analysis of thermal and mechanical properties. The designation of the viscoelastic PUFs was created by adding the prefix “vPU” to the abbreviation of the fat used to obtain the biopolyol that modified the formulation, along with a number corresponding to the amount of biopolyol added.

The obtained viscoelastic foams were subjected to an analysis of their physic-mechanical properties, with particular emphasis on the tests conducted at different ambient temperatures. For basic characterization, the apparent density of the PUFs was measured according to the ISO 845:2006 standard [20].

Thermogravimetric analysis (TGA) was performed on selected samples (containing 0, 5 and 20 wt.% of biopolyol) to determine the thermal stability and degradation mechanism of PUFs. The analyses were carried out using a Q500 thermogravimetric analyzer (TA Instruments, New Castle, DE, USA). Samples were cut from the core region of each foam, avoiding surface zones to minimize the effect of heterogeneity arising from the foaming process. The samples were then ground into irregular fragments weighing between 5 and 9 mg. Measurements were conducted under an inert gas atmosphere (nitrogen) with a flow rate of 90 mL/min to limit oxidation processes.

The temperature program involved heating the samples from room temperature up to 900 °C at a rate of 10 °C/min. Based on the TGA and DTG curves, the temperatures corresponding to 5%, 15%, 50%, and 80% mass loss were determined, denoted as T5%, T15%, T50%, and T80%, respectively. From the DTG curves, characteristic degradation stages were also identified, corresponding to local maxima.

Differential scanning calorimetry (DSC) measurements were performed using a Q200 calorimeter (TA Instruments). The samples weighed between 4 and 5 mg. They were sealed in aluminum pans using a dedicated mechanical press (TA Instruments). Prior to testing, the samples were dried at 40 °C for 24 h. The experiments were carried out under a nitrogen atmosphere at a flow rate of 50 mL/min.

The standard protocol involved three cycles: heating/cooling/heating within the temperature range of −90 to 150 °C, with a constant heating/cooling rate of 10 °C/min. The DSC results analyzed in this study were obtained from the second heating cycle.

Mechanical strength tests were performed using a Zwick/Roell Z005 testing machine (ZwickRoell, Ulm, Germany). For this purpose, foam samples with dimensions of 100 × 100 × 50 mm were placed in a thermal chamber to ensure the desired testing conditions. The viscoelastic foams were tested at −20, 0, 20, and 40 °C. During the measurements, each sample was compressed to 75% of its initial height four times at a compression rate of 100 mm/min. The hardness of the foam material was taken as the compressive stress at 40% foam deformation. Compressive stress measurements were conducted in accordance with PN-EN ISO 3386-1:1997 [21]. Additionally, the collected data were used to calculate the hysteresis Equation (1) and the support factor Equation (2):

(1) $$H=\;\frac{\Delta W}{W_{load}}$$

where ***H***—hysteresis, ∆***W***—the difference in work values during deformation (loading and unloading), ***W_load_***—the work performed during deformation under loading.

(2) $$SF=\frac{F\;\left(65\%\right)}{F\;\left(25\%\right)}$$

where ***SF***—support factor, ***F*** (65%)—compressive strength at 65% deformation of the tested sample, ***F*** (25%)—compressive strength at 25% deformation of the tested sample.

## 3. Results

**Figure 1:**
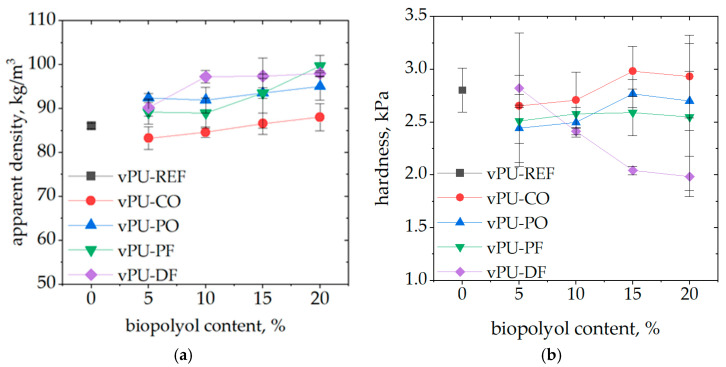
Apparent density (**a**), hardness (**b**) of viscoelastic PUFs prepared with the addition of biopolyols.

The first analysis performed was the determination of the apparent density of the foamed materials, which can significantly influence their mechanical properties [22]. Figure 1a presents the effect of the biopolyol content on the apparent density of the obtained foams. A general trend of increasing foam apparent density with increasing biopolyol content up to 20 wt.% was observed, amounting to approximately 10 kg/m^3^ compared to the reference foam without biopolyol, which had an apparent density of 86 kg/m^3^. The foams prepared with coconut oil-based biopolyol, for which the apparent density initially slightly decreased and then increased only marginally above that of the reference foam were only an exception. This behavior may be attributed to the relatively low viscosity of the bio-CO, which can facilitate improved foaming efficiency and promote the formation of larger cells, thereby counteracting the density increase typically associated with higher biopolyol content. Figure 1b shows the compressive strength at 40% deformation (hardness) measured at room temperature, which is considered the foam hardness.

Commercial viscoelastic polyurethane foams typically used in furniture and bedding applications exhibit apparent densities in the range of approximately 40–80 kg/m^3^ and low hardness values. The hardness of commercial viscoelastic foams is most commonly reported in the range of approximately 1–3 kPa, although values exceeding 10 have also been described [23]. The apparent density and hardness values obtained for the foams investigated in this study fall within or close to these typical ranges, indicating that the developed biobased viscoelastic foams are comparable to conventional commercial materials in terms of their basic structural and mechanical characteristics. The analysis of apparent density and hardness of PUFs demonstrated clear differences between the tested foams, reflecting the influence of the applied biopolyols on the structural characteristics of the materials. Although all samples were produced within a relatively narrow apparent density range (approximately 83–100 kg/m^3^), the measured hardness values varied noticeably across the formulations. In most cases, an increase or decrease in apparent density did not directly correspond to proportional changes in foam hardness, indicating that the internal structure and the specific interactions from the natural additives played a dominant role in shaping the mechanical response. In the case of vPU-DF, the data reveal an atypical trend compared to conventional PUFs, namely a systematic decrease in hardness with increasing apparent density. Such behavior suggests that factors other than apparent density—most likely the chemical nature of the applied biopolyols, their plasticizing action, and the resulting modifications in cell morphology—play a dominant role in governing the mechanical response of the modified foams. Importantly, the biopolyols used in the formulations exhibited a distinct plasticizing effect, which contributed to a reduction in foam hardness by increasing the mobility of polymer chains within the soft segments of the polyurethane network. This effect was strongly dependent on the molecular architecture of the biopolyols—particularly the length and flexibility of their hydrocarbon chains, the presence of dangling groups, and their functionality—which influenced both the crosslink density and physical interactions between chains [24,25].

**Figure 2:**
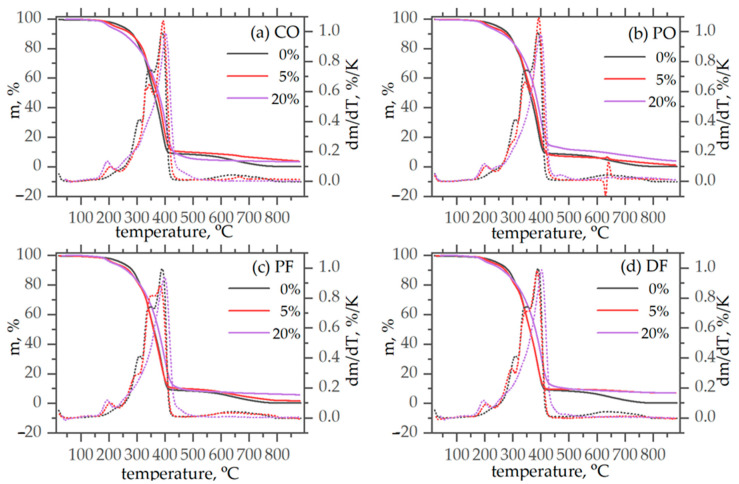
TG (continuous lines) and DTG (dotted lines) curves of the vPU-REF and foams modified by biopolyols: (**a**) vPU-CO; (**b**) vPU-PO; (**c**) vPU-PF; (**d**) vPU-DF.

To evaluate the effect of modification by biopolyols on the thermal stability of foams, thermogravimetric (TG) analysis was performed for the reference sample (vPU-REF) and for the samples containing the extreme contents, i.e., 5 wt.% and 20 wt.% of each type of biopolyol (Figure 2a–d). TG analysis was limited to these compositions to clearly demonstrate the influence of bio-polyol incorporation on thermal stability, as samples with intermediate bio-polyol contents exhibited the same degradation trends. The focus of the present study is on the temperature-dependent functional performance of the foams rather than on detailed compositional analysis.

The degradation process of polyurethane elastomers and foams is a very complicated process depending strongly on the chemical composition of the systems as well as in their micromorphology [26]. The reference, petrochemical-based system, of this study, in general follows the standard pattern observed in degradation of polyurethane foams under inert conditions [27,28,29,30]. Starting from low temperatures, a mass loss around 230 °C should be attributed to evaporation of small volatile molecules remaining in the system upon synthesis (Figure 2). This is followed by the main degradation step centered around 350 °C. The DTG signal reveals that this step is a complex one, with three distinct components showing maximum degradation rates at 306, 346, and 386 °C. The first steps should be associated in the literature to scission of urethane, producing isocyanate and polyol segments [29]. The second and third steps should be associated with the degradation of the polyol segments at higher temperatures into aliphatic ether alcohols and other volatile products [29], forming also a residue similar to the ash produced in the combustion process [29]. The emergence of the two distinct peaks should be attributed to the three different polyols in the mixture. The complexity of the system, however, does not allow for further development for hypotheses at this stage.

Modification by all biopolyols has the same quite interesting qualitative effects. Starting from low temperatures, the peak associated with small volatile molecules intensifies and migrates to lower temperatures with increasing biopolyols content. This is not surprising. The biopolyols derived from natural fats may contain additional, non-reactive small fragments of oils which evaporate at lower temperatures.

The main degradation step migrates to somewhat higher temperatures indicating, at a first glance, a moderate overall improvement in thermal stability in the modified foams. A more detailed look at the DTG signals, however, reveals a more complex behavior. Namely, the first component (centered at 306 °C in the reference system), migrates to lower temperatures, weakens and broadens. This should be associated with higher lability of the urethane bonds formed with the biopolyols and increased heterogeneity in the molecular level. This is consistent with the substitution of relatively well defined polyols with low polydispersity with complex mixtures of compounds having even multimodal molar mass distributions [17]. In addition to that, biopolyols, with average molar mass in the order of 500 substitute in part a polyol of molar mass of the order of 5000 (P3), clearly increasing heterogeneity. The second step, associated with a first degradation of the polyol backbones, at 20 wt.% modification reduces to a shoulder on the main peak. This should be attributed to its broadening and migration to higher temperatures, which is also consistent with homogenization of the system on the molecular level. This is corroborated by a slight migration of the peak towards higher temperatures, for 5 wt.% modification with the polyols derived from animal fats.

The aforementioned observations are also reflected in the temperatures at mass loss 5%, 15%, 50%, and 80% (Table 3). All biopolyol-modified foams exhibited lower initial decomposition temperatures (T5%) compared to the reference vPU-REF (246.4 °C), due to the release of volatile or thermally labile fractions introduced by the biopolyols. Despite the lower T5%, the T50% values remained comparable or slightly higher for foams containing 20 wt.% bio-polyol (e.g., vPU-PO-20, T50% = 380.1 °C), suggesting that bio-polyols, while promoting early mass loss, do not adversely affect and may even improve the thermal stability of the foams at higher temperatures, as described qualitatively in the previous paragraph.

The high temperature effect associated provisionally with volatilization of the residue is strongly suppressed by modification. Notably, the final residue at 850 °C increased substantially for several formulations (e.g., vPU-PF-20 = 6.01%, vPU-DF-5 = 7.16%), which indicates enhanced char formation likely due to fatty-acid-derived structures favoring carbonaceous residue upon pyrolysis. These observations imply that the effect of biopolyols is twofold: introduction of easily volatilized species (lower T5%) and promotion of char-forming pathways (higher residue), with the magnitude strongly dependent on both biopolyol chemical nature and its content in foams.

**Figure 3:**
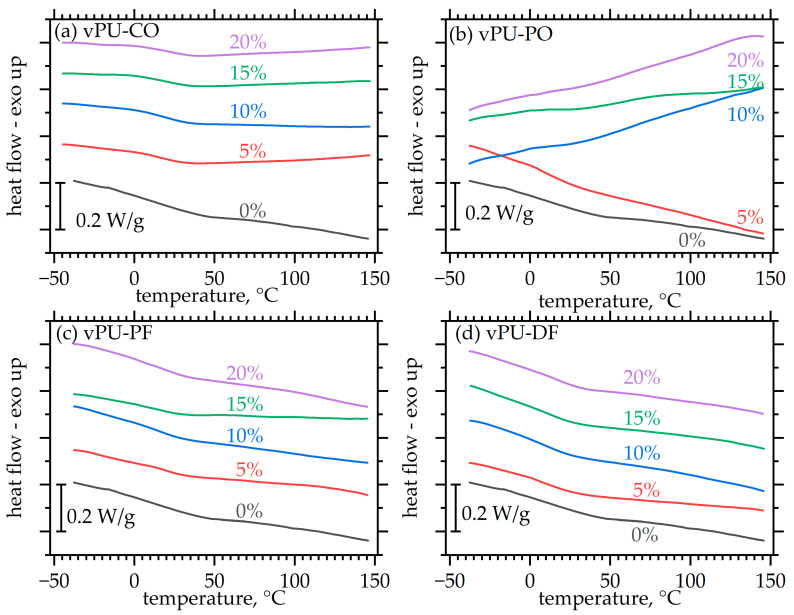
DSC curves recorded with all materials under investigation. The curves have been translated vertically for clarity. (**a**) vPU-CO, (**b**) vPU-PO, (**c**) vPU-PF, (**d**) vPU-DF.

The samples of the obtained foams were also subjected to DSC analysis. Figure 3 shows the curves for the tested viscoelastic PUFs.

The main feature of the DSC curves is a very weak and broad step, spanning the temperature range −20 to 60 °C (Figure 3). This curve morphology is quite common in polyurethanes, and for rigid ones, it is observed at temperatures usually above 100 °C [31,32,33]. In other works on viscoelastic foams, the authors detected a low temperature glass transition at temperatures as low as −60 °C with a very broad peak at above-ambient temperatures, attributed vaguely to the transformation of order in hard domains [4,34]. Experience mainly in elastomeric polyurethane materials, where a larger body of research is available, shows that such behaviors in the discussed region are related to the hard segments, and they are associated with their softening/glass transition which often manifests itself as a peak associated with enthalpy relaxation [35]. In any case, this is a very challenging question as it also largely depends on the level of ordering within hard microdomains. Summing up, we believe that what is seen here is the glass transition of the foams associated with their hard segments.

Glass transition, and especially its dynamic manifestation, is a key phenomenon of PUFs largely defining their functionality as memory foams [36]. The effect of humidity in the dynamics glass transition and subsequently to performance of materials has also been described in detail [37]. The thermal transitions in these systems are centered around 10–20 °C, which makes them promising candidates for memory foams. They are likely to exhibit recovery times of a few seconds at temperatures close to those relevant for bedding applications and direct contact with the human body.

**Figure 4:**
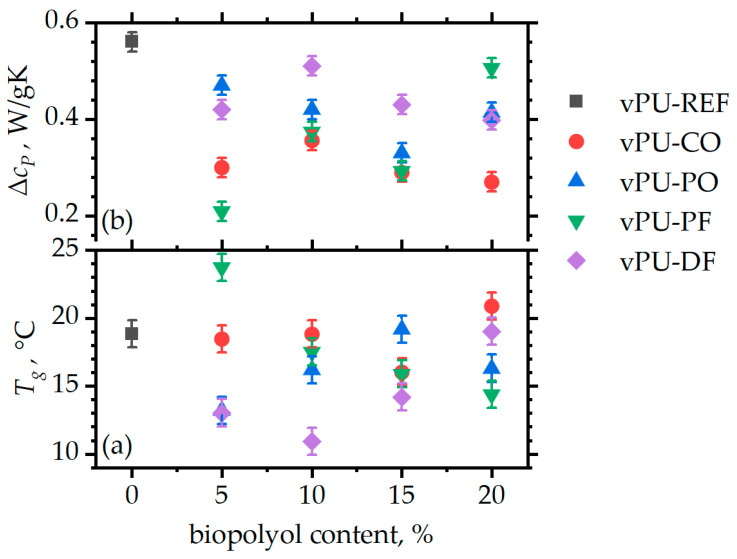
(**a**) Midpoint glass transition temperature *T_g_*; (**b**) heat capacity change Δ*c_p_* at *T_g_*.

The unusually broad form of the glass transition steps, along with the weak signal, leads to quite high uncertainties in calculating glass transition temperatures *T_g_* and heat capacity changes. Nevertheless, it can be observed (Figure 4a) that midpoint values remain mostly in the 10–20 °C range, suitable for memory foam application. For most natural fats, foams seem to have a glass transition temperature somewhat lower than that of the matrix indicating a plasticizing action. However, the behavior of foams does not show consistent trends, and is known to vary with plenty of parameters, including degree of phase separation, humidity, etc., therefore any further comment would be largely a speculation. Similarly, the intensity of the glass transition step as quantified by the heat capacity change Δ*c_p_* seems to be somewhat smaller than the reference material; however, for the same fat, dependence on foam composition is weak.

**Figure 5:**
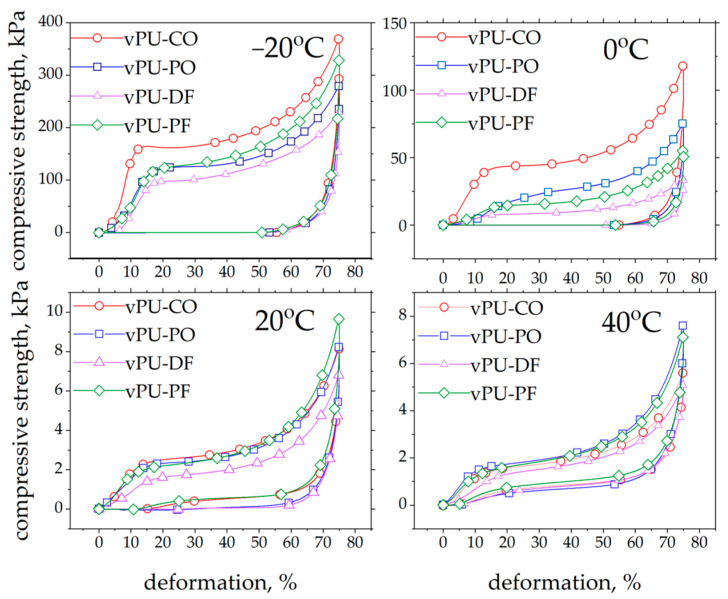
Hysteresis loops for foams containing 15 wt.% of bio-CO, bio-PO, bio-PF, and bio-DF at different test temperatures.

The next stage of the study involved evaluating the typical mechanical properties of the foams obtained within the intended application temperature range, i.e., from −20 °C to +40 °C. For this purpose, hysteresis loops were determined for foams compressed to 75% of their initial height. During compression, the stress was measured both during loading and unloading of the foams. Figure 5 presents example hysteresis loops obtained at different temperatures for foams modified with 15 wt.% biopolyols.

It was observed that, as the temperature increased, the area of the hysteresis loop became smaller and the strength range decreased significantly. The data obtained from the hysteresis loops were then used to determine further properties of the analyzed foams. The results clearly indicate that testing temperature has a significant effect on the hardness of the viscoelastic PUFs investigated.

**Figure 6:**
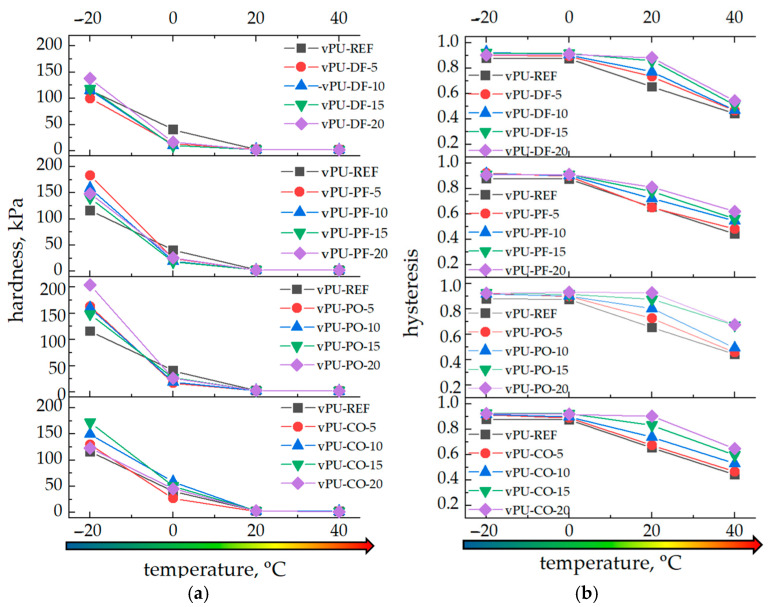
The effect of temperature variation on the hardness (**a**) and hysteresis (**b**) of viscoelastic PUFs.

Figure 6a presents the results of hardness measurements of viscoelastic PUFs as a function of biopolyol content in the polyurethane composition and the testing temperature (−20, 0, 20, and 40 °C). The detailed results of the mechanical tests are included in the Supplementary (Tables S1–S4).

The results demonstrate a strong temperature dependence: with increasing temperature, the hardness of all foams decreases. At −20 °C, the average hardness reaches approximately 145 kPa, while at 40 °C it decreases to around 2 kPa. This confirms that the compressive compliance of the polyurethane materials increases significantly at elevated temperatures. At 0 °C the hardness is approximately 50 kPa, whereas at −20 °C it ranges from 100 to 200 kPa depending on the type and amount of biopolyol.

Clear differences were observed between the foams modified with different biopolyols. The fats used to produce the biopolyols differ in their fatty-acid composition, which directly influences the structure and performance of the obtained foams. A detailed analysis of their lipid profiles was presented in our previous work [17]. Coconut oil is rich in short saturated fatty acids, palm oil and pork fat contain mainly long-chain saturated fatty acids, while duck fat has a high content of unsaturated fatty acids.

These structural differences strongly affect the materials’ behavior at low temperatures. Foams based on bio-PO and bio-PF were generally harder at moderate temperatures due to the presence of long saturated chains. However, at −20 °C the vPU-CO foams exhibited the highest hardness among all systems, even though they were not the most rigid at room temperature. This effect results from the large amount of short saturated fatty acids in CO. At temperatures below the glass transition, these short chains have significantly slower molecular mobility, causing a strong physical stiffening of the vPU-CO foams. In contrast, vPU-DF foams remain the softest at low temperatures because the unsaturated fatty acids act as internal plasticizers.

At 0 °C, the foams are in the transition zone between the glassy and viscoelastic states, with hardness values ranging from 10 to 60 kPa. The vPU-CO and vPU-PO foams retained higher mechanical strength, suggesting a more stable network structure. The vPU-DF foams showed lower hardness but better damping properties, which may be useful for impact-absorbing applications.

At 20 °C (typical service conditions), all foams displayed stable viscoelastic behavior, with hardness values between 2 and 3 kPa, similar to commercial memory foams. Increasing the biopolyol content slightly reduced hardness and improved comfort-related properties. The vPU-PO and vPU-PF foams showed slightly higher hardness, suggesting better load-bearing capacity, whereas vPU-CO and vPU-DF exhibited higher damping. At 40 °C, all foams became softer due to increased chain mobility, with hardness values of about 1.5–2.0 kPa. The most pronounced softening occurred in vPU-CO and vPU-DF, which confirms more plasticizing efects bio-CO and bio-DF at elevated temperatures.

The mechanical behavior of viscoelastic PUFs is strongly temperature-dependent because of the significant changes in chains mobility occurring as the sample crosses the glass transition region. At −20 °C, the foam is in a glassy state, leading to hardness values above 100 kPa and behavior characteristic of semi-rigid foams. At 0 °C, the materials begin to regain mobility, and hardness decreases to 10–60 kPa. At 20 °C, all foams achieve optimal viscoelastic performance, providing a combination of support and slow elastic recovery. At 40 °C, further softening occurs, enhancing comfort and pressure distribution, which is beneficial for applications involving direct human contact.

This strong temperature responsiveness is typical for viscoelastic PUFs. At 20 °C and 40 °C, the bio-based foams showed stable and comfortable viscoelastic behavior, making them suitable for applications in furniture and bedding. However, at 0 °C and especially at −20 °C, the hardness of the materials increased sharply, causing the foams to become too hard for comfortable use. As a result, these materials may not be suitable for all applications, particularly those requiring good comfort performance at low temperatures.

The DSC curves of all examined foams show a very broad and weakly defined step corresponding to the glass transition, with glass-transition temperatures (*T_g_*) mostly falling within the range of 10–20 °C (Figure 3). The obtained DSC results provide a useful reference for interpreting the temperature-dependent changes in the mechanical properties of the foams. The proximity of the *T_g_* range to the application temperatures explains the pronounced variations in mechanical behavior observed between −20 and 40 °C. When the testing temperature is below *T_g_* (e.g., −20 °C), chain mobility is restricted, and hardness and compressive strength increase sharply (with observed hardness values of ~100–200 kPa). In contrast, at temperatures above or near *T_g_* (20–40 °C), the soft segments regain mobility, and the foams exhibit properties typical (hardness ~1.5–3 kPa) for PUFs.

Figure 6b presents the hysteresis values as a function of biopolyol type, content in the polyurethane composition and test temperature (−20, 0, 20, and 40 °C). The results indicate that both temperature and biopolyol type and content affect the hysteresis of the foams. With increasing ambient temperature, hysteresis decreases. Conversely, with increasing biopolyol content, hysteresis in the case of all biopolyol types rises across all tested temperatures. A lower hysteresis value corresponds to a faster recovery of the sample to its original shape after load removal. Thus, the incorporation of biopolyols delays the recovery of the deformed samples. At low temperatures, hysteresis exceeds 0.9 and is approximately the same at 0 and −20 °C. At 20 °C, its value decreases to about 0.8, and at 40 °C it further decreases to approximately 0.6. The pronounced effect of test temperature has a significant impact on the performance of viscoelastic foams, which poses challenges in extending their practical application range. Nevertheless, the incorporation of biopolyols was found to mitigate property changes at 0 °C compared to the reference foams, suggesting improved stability under moderate sub-ambient conditions. At −20 °C, however, this effect becomes insignificant, indicating that the benefits of biopolyol incorporation are limited to a narrower temperature window.

**Figure 7:**
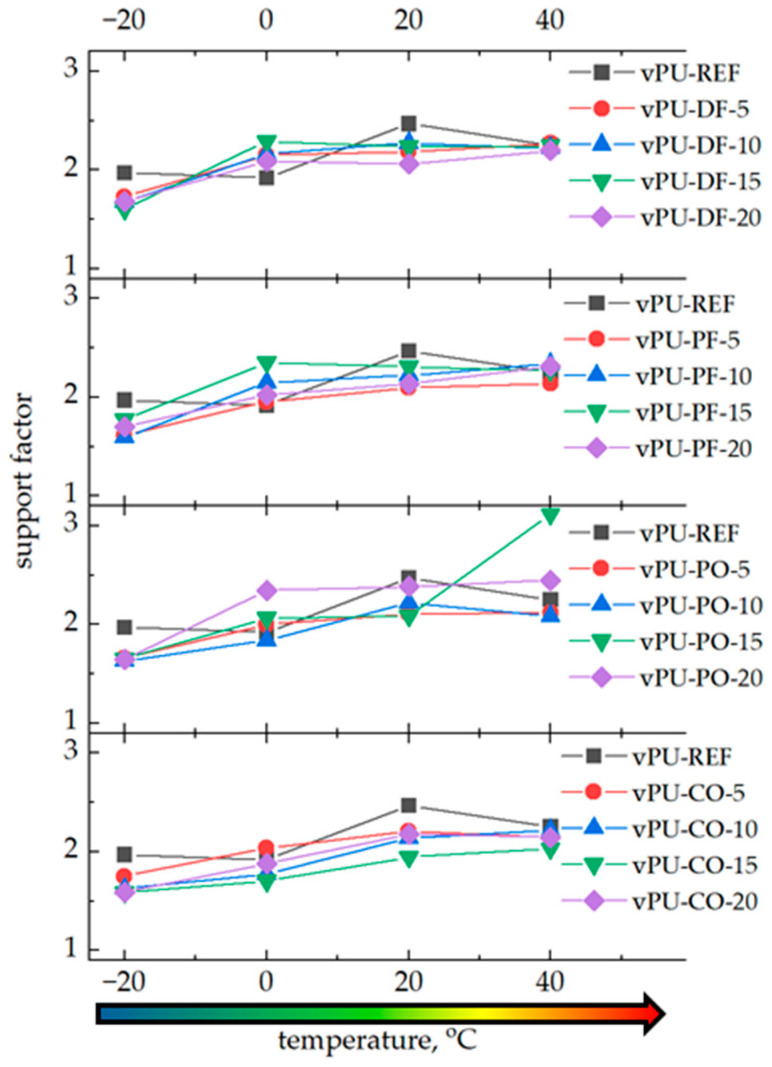
The impact of temperature and the biopolyol content in polyol premix on the support factor of PUFs.

Figure 7 illustrates the changes in one of the key indicators reflecting the comfort of using a given foam material, namely the support factor. The support factor is calculated as the ratio of the foam’s compressive strength measured at 65% and 25% deformation. It was also observed that ambient temperature affects the value of this parameter. At subzero temperatures, the comfort factor is approximately 2 for the reference foam and below 2 for the foams modified with biopolyols. As the testing temperature increases, the comfort factor rises to around 2.5. Materials are generally considered most comfortable when the support factor is around 3 [3]. This indicates that they feel soft at first touch, yet provide adequate support under higher loads. In the analyses conducted at 0 °C or −20 °C, the support factor was approximately 2. However, comfort perception in this case is limited, as the material’s hardness increases several dozen times at 0 °C and up to one hundred times at −20 °C compared to material hardness under room conditions. Sitting or lying on a mattress with such high hardness would hardly be considered comfortable, despite the relatively favorable support factor value.

## 4. Conclusions

In this study, the effect of introducing biopolyols derived from coconut and palm oils as well as duck and pork fats to the polyurethane system on the physicomechanical properties of viscoelastic polyurethane foams at different temperatures was investigated. The research was conducted at temperatures of −20, 0, 20, and 40 °C and with various biopolyol contents in the polyurethane composition (5, 10, 15, and 20 wt.% in polyol premix). Based on the obtained results, it can be concluded that both the content of biopolyol and its type, as well as the test temperature influence the properties of the modified viscoelastic foams.

Thermogravimetric analysis showed that the introduction of biopolyols affects the manner and intensity of polyurethane foam degradation compared to the reference sample containing only petrochemical polyol. In all systems containing biopolyol, a decrease in the initial degradation temperature was observed, although the main degradation step seemed to move at slightly higher temperatures.

The glass transition of the foams is in a range appropriate for application of the foams in furnitures (somewhat below room and body temperature), and the introduction of the biopolyols does not seem to have any significant effect on this parameter, as compared to the reference system.

As expected, the mechanical response of the foams strongly depended on the testing temperature. At low temperatures (−20 °C), all foams exhibited a significant increase in hardness (up to 200 kPa) compared to room-temperature values (~2–3 kPa), indicating restricted chain mobility and the dominance of the hard segment domains. Conversely, at elevated temperatures (40 °C), the materials became much softer (hardness ~1.5–2.5 kPa), reflecting enhanced mobility of the soft segments.

The results clearly demonstrate that the type and structure of the applied biopolyol play a crucial role in defining the viscoelastic behavior of polyurethane foams. Biopolyols derived from more unsaturated fats (DF) act as internal plasticizers, yielding softer and more energy-absorbing materials, while biopolyols with long and saturated chains (PF and PO) enhance stiffness and structural stability.

Importantly, all foams containing up to 20 wt.% biopolyol exhibited mechanical and comfort properties comparable to those of commercial viscoelastic polyurethane foams, confirming the possibility of partially substituting petrochemical polyols with bio-based alternatives without compromising performance.

## Figures and Tables

**Table 1:** Biopolyol characteristics.

Biopolyol	OHv, mg KOH/g	% H_2_O, %	Viscosity, mPa·s
bio-PO	330	0.3	110
bio-CO	380	0.2	55
bio-PF	350	0.2	80
bio-DF	320	0.2	65

**Table 2:** Polyurethane foam formulations.

Foam Symbol	Components, g								
	P1	P2	P3	Biopolyol	Catalyst	Surfactant	H_2_O	DEG	Isocyanate
vPU-REF	40.0	20.0	40.0	-	1.0	0.8	2.5	3.0	45.1
vPU-PO-5	37.5	20.0	37.5	5.0	1.0	0.8	2.5	3.0	45.8
vPU-CO-5	37.5	20.0	37.5	5.0	1.0	0.8	2.5	3.0	46.1
vPU-PF-5	37.5	20.0	37.5	5.0	1.0	0.8	2.5	3.0	46.0
vPU-DF-5	37.5	20.0	37.5	5.0	1.0	0.8	2.5	3.0	45.8
vPU-PO-10	35.0	20.0	35.0	10.0	1.0	0.8	2.5	3.0	46.5
vPU-CO-10	35.0	20.0	35.0	10.0	1.0	0.8	2.5	3.0	47.2
vPU-PF-10	35.0	20.0	35.0	10.0	1.0	0.8	2.5	3.0	46.9
vPU-DF-10	35.0	20.0	35.0	10.0	1.0	0.8	2.5	3.0	46.6
vPU-PO-15	32.5	20.0	32.5	15.0	1.0	0.8	2.5	3.0	47.3
vPU-CO-15	32.5	20.0	32.5	15.0	1.0	0.8	2.5	3.0	48.3
vPU-PF-15	32.5	20.0	32.5	15.0	1.0	0.8	2.5	3.0	47.8
vPU-DF-15	32.5	20.0	32.5	15.0	1.0	0.8	2.5	3.0	47.4
vPU-PO-20	30.0	20.0	30.0	20.0	1.0	0.8	2.5	3.0	48.0
vPU-CO-20	30.0	20.0	30.0	20.0	1.0	0.8	2.5	3.0	49.4
vPU-PF-20	30.0	20.0	30.0	20.0	1.0	0.8	2.5	3.0	48.7
vPU-DF-20	30.0	20.0	30.0	20.0	1.0	0.8	2.5	3.0	48.2

**Table 3:** Thermal decomposition data of vPU foams with various biopolyols.

Sample	T_5%_ [°C]	T_15%_ [°C]	T_50%_ [°C]	T_80%_ [°C]	Residue at 850 °C (%)
vPU-REF	246.4	299.6	360.1	394.6	0.35
vPU-PO-5	220.4	297.2	366.6	399.9	1.55
vPU-PO-20	209.4	295.7	380.1	413.6	4.38
vPU-PF-5	217.1	290.9	358.5	394.1	1.84
vPU-PF-20	211.3	290.6	376.1	410.5	6.01
vPU-DF-5	228.6	291.4	360.0	394.5	7.16
vPU-DF-20	211.9	292.2	380.0	413.7	7.11
vPU-CO-5	222.9	302.7	370.0	402.6	4.24
vPU-CO-20	202.4	282.7	376.8	411.4	3.45

## Data Availability

The raw data supporting the conclusions of this article will be made available by the authors on request.

## Supplementary Materials

The following supporting information can be downloaded at: https://www.mdpi.com/article/10.3390/polym18020174/s1, Table S1: Hardness, hysteresis and support factor of viscoelastic foams at 40 °C; Table S2: Hardness, hysteresis and support factor of viscoelastic foams at 20 °C; Table S3. Hardness, hysteresis and support factor of viscoelastic foams at 0 °C; Table S4. Hardness, hysteresis and support factor of viscoelastic foams at −20 °C.
