# Supplementary material for: Bio-Based Viscoelastic Polyurethane Foams: Functional Behavior Across Application Temperatures
Source: Polymers (Basel). 2026 Jan 8;18(2):174. doi: 10.3390/polym18020174 (PMC12846099; doi:10.3390/polym18020174)
Supplement: Supplementary file 1 [file polymers-18-00174-s001.zip › polymers-4058793-supplementary.pdf]

Table S1. Hardness, hysteresis and support factor of viscoelastic foams at 40°C.

	40°C					
	hardness (kPa)	σ	hysteresis	σ	support factor	σ
vPU-REF	2.12	0.100	0.44	0.007	2.2	0.01
vPU-CO-5	2.05	0.040	0.46	0.020	2.1	0.07
vPU-CO-10	1.93	0.140	0.53	0.010	2.2	0.15
vPU-CO-15	1.91	0.120	0.60	0.030	2.0	0.01
vPU-CO-20	1.76	0.200	0.65	0.010	2.1	0.06
vPU-DF-5	2.15	0.070	0.47	0.010	2.3	0.14
vPU- DF-10	2.08	0.007	0.47	0.006	2.2	0.05
vPU-DF-15	1.74	0.030	0.51	0.009	2.2	0.05
vPU-DF-20	1.48	0.110	0.54	0.010	2.2	0.04
vPU-PO-5	2.06	0.110	0.46	0.010	2.1	0.05
vPU- PO-10	1.90	0.070	0.49	0.002	2.1	0.01
vPU-PO-15	2.17	0.010	0.67	0.070	3.1	1.05
vPU-PO-20	1.97	0.150	0.68	0.010	2.4	0.19
vPU-PF-5	2.19	0.170	0.48	0.020	2.1	0.02
vPU- PF-10	2.46	0.350	0.55	0.050	2.3	0.11
vPU-PF-15	1.95	0.230	0.56	0.040	2.3	0.04
vPU-PF-20	1.98	0.250	0.62	0.040	2.3	0.17

Table S2. Hardness, hysteresis and support factor of viscoelastic foams at 20°C.

	20°C					
	hardness (kPa)	σ	hysteresis	σ	Suport factor	σ
vPU-REF	2.80	0.210	0.65	0.010	2.5	0.07
vPU-CO-5	2.65	0.010	0.67	0.020	2.2	0.07
vPU-CO-10	2.71	0.265	0.74	0.010	2.1	0.11
vPU-CO-15	2.98	0.237	0.83	0.040	1.9	0.02
vPU-CO-20	2.93	0.392	0.90	0.007	2.2	0.12
vPU-DF-5	2.82	0.521	0.74	0.010	2.2	0.06
vPU- DF-10	2.41	0.033	0.77	0.001	2.3	0.03
vPU-DF-15	2.04	0.040	0.86	0.010	2.2	0.05
vPU-DF-20	1.99	0.190	0.88	0.010	2.1	0.05
vPU-PO-5	2.44	0.322	0.73	0.010	2.1	0.09
vPU- PO-10	2.50	0.140	0.80	0.010	2.2	0.07
vPU-PO-15	2.77	0.133	0.88	0.001	2.1	0.04
vPU-PO-20	2.70	0.280	0.93	0.005	2.4	0.20
vPU-PF-5	2.51	0.433	0.65	0.010	2.1	0.01
vPU- PF-10	2.58	0.000	0.72	0.009	2.2	0.08

vPU-PF-15	2.59	0.220	0.78	0.040	2.3	0.01
vPU-PF-20	2.55	0.695	0.81	0.020	2.1	0.03

Table S3. Hardness, hysteresis and support factor of viscoelastic foams at 0°C.

	0°C					
	hardness (kPa)	σ	hysteresis	σ	Suport factor	σ
vPU-REF	40.71	0.070	0.88	0.005	1.9	0.04
vPU-CO-5	26.85	0.890	0.89	0.003	1.9	0.00
vPU-CO-10	58.43	0.160	0.90	0.000	2.0	0.00
vPU-CO-15	50.21	2.790	0.92	0.003	1.8	0.02
vPU-CO-20	45.37	4.720	0.92	0.002	1.7	0.02
vPU-DF-5	13.39	2.230	0.89	0.001	2.2	0.06
vPU- DF-10	10.06	0.490	0.91	0.002	2.2	0.07
vPU-DF-15	9.82	0.010	0.92	0.005	2.3	0.11
vPU-DF-20	16.17	0.430	0.91	0.001	2.1	0.07
vPU-PO-5	16.61	4.210	0.90	0.002	2.0	0.04
vPU- PO-10	18.74	2.990	0.90	0.001	1.8	0.01
vPU-PO-15	26.85	4.410	0.92	0.000	2.1	0.02
vPU-PO-20	25.97	2.780	0.93	0.004	2.3	0.19
vPU-PF-5	25.18	0.090	0.90	0.001	2.0	0.05
vPU- PF-10	19.39	3.610	0.90	0.001	2.2	0.06
vPU-PF-15	18.37	0.640	0.91	0.002	2.4	0.08
vPU-PF-20	26.21	3.870	0.91	0.003	2.0	0.05

Table S4. Hardness, hysteresis and support factor of viscoelastic foams at -20°C.

	-20°C					
	hardness (kPa)	σ	hysteresis	σ	Suport factor	σ
vPU-REF	116.00	8.390	0.88	0.010	2.2	0.32
vPU-CO-5	129.20	1.270	0.91	0.001	2.0	0.01
vPU-CO-10	149.50	11.020	0.92	0.001	1.7	0.05
vPU-CO-15	172.20	9.190	0.93	0.001	1.6	0.06
vPU-CO-20	122.64	5.230	0.93	0.001	1.6	0.06
vPU-DF-5	99.36	4.230	0.91	0.006	1.7	0.14
vPU- DF-10	115.63	24.930	0.93	0.001	1.7	0.02
vPU-DF-15	118.30	0.000	0.92	0.000	1.6	0.00
vPU-DF-20	138.06	5.690	0.90	0.001	1.7	0.04
vPU-PO-5	162.80	6.370	0.92	0.001	1.7	0.01
vPU- PO-10	160.10	7.270	0.92	0.002	1.6	0.02
vPU-PO-15	148.50	14.500	0.93	0.002	1.7	0.06

vPU-PO-20	204.10	8.300	0.93	0.001	1.6	0.00
vPU-PF-5	183.00	7.230	0.92	0.001	1.6	0.03
vPU-PF-10	158.90	14.010	0.92	0.004	1.6	0.05
vPU-PF-15	140.10	2.000	0.91	0.000	1.8	0.00
vPU-PF-20	148.29	4.800	0.91	0.003	1.7	0.04
